# Pharmacokinetics and Pharmacodynamics of Fosravuconazole, Itraconazole, and Hydroxyitraconazole in Sudanese Patients With Eumycetoma

**DOI:** 10.1093/infdis/jiaf279

**Published:** 2025-05-28

**Authors:** Wan-Yu Chu, Ahmed H Fahal, Eiman Siddig Ahmed, Sahar Mubarak Bakhiet, Osama Elhadi Bakhiet, Lamis Ahmed Fahal, Abubakar Ahmed Mohamed, El Sammani Wadaa Mohamedelamin, Mustafa El Nour Bahar, Hadil Yassir Attalla, Emmanuel Edwar Siddig, Najwa A Mhmoud, Ahmed Mudawi Musa, Peelen Oyieko, Thaddaeus Egondi, Roger J Brüggemann, Katsura Hata, Nathalie Strub-Wourgaft, Fabiana Alves, Borna A Nyaoke, Eduard E Zijlstra, Thomas P C Dorlo

**Affiliations:** Department of Pharmacy, Uppsala University, Uppsala, Sweden; Department of Pharmacy and Pharmacology, Netherlands Cancer Institute, Amsterdam, The Netherlands; Mycetoma Research Center, University of Khartoum, Khartoum, Sudan; Mycetoma Research Center, University of Khartoum, Khartoum, Sudan; Mycetoma Research Center, University of Khartoum, Khartoum, Sudan; Institute of Endemic Diseases, University of Khartoum, Khartoum, Sudan; Mycetoma Research Center, University of Khartoum, Khartoum, Sudan; Mycetoma Research Center, University of Khartoum, Khartoum, Sudan; Mycetoma Research Center, University of Khartoum, Khartoum, Sudan; Mycetoma Research Center, University of Khartoum, Khartoum, Sudan; Mycetoma Research Center, University of Khartoum, Khartoum, Sudan; Mycetoma Research Center, University of Khartoum, Khartoum, Sudan; Mycetoma Research Center, University of Khartoum, Khartoum, Sudan; Mycetoma Research Center, University of Khartoum, Khartoum, Sudan; Mycetoma Research Center, University of Khartoum, Khartoum, Sudan; Institute of Endemic Diseases, University of Khartoum, Khartoum, Sudan; Drugs for Neglected Diseases Initiative, Nairobi, Kenya; Drugs for Neglected Diseases Initiative, Nairobi, Kenya; Department of Pharmacy, Radboud University Medical Center-Canisius Wilhelmina Ziekenhuis (Radboudumc-CWZ Center) Expertise for Mycology and Radboudumc Institute for Medical Innovation, Radboud University Medical Center, Nijmegen, The Netherlands; Global Health Research Section, Eisai Co, Ltd, Ibaraki, Japan; Drugs for Neglected Diseases Initiative, Geneva, Switzerland; Drugs for Neglected Diseases Initiative, Geneva, Switzerland; Drugs for Neglected Diseases Initiative, Nairobi, Kenya; Drugs for Neglected Diseases Initiative, Geneva, Switzerland; Department of Pharmacy, Uppsala University, Uppsala, Sweden

**Keywords:** pharmacokinetics, pharmacodynamics, fosravuconazole, itraconazole, mycetoma

## Abstract

**Background:**

The first clinical trial on eumycetoma was recently conducted in Sudan, comparing oral fosravuconazole, prodrug of active ravuconazole, with the standard-of-care oral itraconazole. Building on this trial, the present study aimed to characterize the pharmacokinetics-pharmacodynamics (PK-PD) of ravuconazole, itraconazole, and hydroxyitraconazole in patients with eumycetoma and guide selection of either a 200-mg or 300-mg dose of fosravuconazole.

**Methods:**

Nonlinear mixed-effects modeling was used to develop population PK models in 52 patients receiving 3 daily loading doses followed by weekly fosravuconazole (200 mg or 300 mg) or twice-daily itraconazole (total 400 mg), both over 12 months. Attainment of the in vitro 90% minimum inhibitory concentration (MIC_90_) for *Madurella mycetomatis* was assessed, and the relationships between drug exposure, lesion size reduction, and complete cure were evaluated.

**Results:**

Ravuconazole PK followed a 2-compartment model with Michaelis-Menten elimination and a 63% (95% confidence interval, 38%–90%) bioavailability increase during the loading phase, leading to 75% higher exposure for a 50% dose increase. Itraconazole and hydroxyitraconazole were modeled jointly, with autoinhibition of itraconazole metabolism. Free ravuconazole remained above the MIC_90_ throughout the entire 12-month treatment period, while free itraconazole never reached the MIC_90_. Despite a large range in antifungal exposure, no significant relationships were found between drug exposure and lesion size reduction or complete cure, indicating no additional benefit of 300 mg over 200 mg fosravuconazole.

**Conclusions:**

Ravuconazole and itraconazole showed nonlinear clearance with no clear exposure-response relationship. The 200 mg fosravuconazole dose is preferred for future use over 300 mg, as it lowers pill burden and enhances cost-effectiveness.

**Clinical Trials Registration**. NCT03086226.

## Teaser

This first pharmacokinetics study in patients with eumycetoma demonstrated nonlinear clearance of both ravuconazole and itraconazole. No clear exposure-response relationship was found, supporting the use of 200 mg fosravuconazole over 300 mg to reduce pill burden and enhance cost-effectiveness.

Mycetoma is a neglected tropical skin disease cause by either bacteria (actinomycetoma) or fungi (eumycetoma). It typically presents as a painless subcutaneous mass with multiple sinuses that discharge grains containing the causative organism [1]. Eumycetoma, most commonly caused by the fungus *Madurella mycetomatis,* often develops following minor trauma such as a thorn prick, and mainly affects young adults in impoverished communities in low- and middle-income countries [2]. Limited access to medical care and a lack of effective drugs often result in late-stage presentations with frequent recurrences, making amputation the only viable option.

Current treatment for eumycetoma combines surgery with at least 12 months of twice-daily oral itraconazole capsules [3]. However, the effectiveness of itraconazole is unclear, as no clinical trial has been conducted [4, 5]. In addition, its high cost and limited availability in endemic areas lead to reliance on ketoconazole, a drug with risks of adrenal and liver toxicity. Fosravuconazole, a triazole antifungal agent that is approved in Japan for the treatment of onychomycosis [6], was recently evaluated for the treatment of eumycetoma in the first-ever clinical trial (NCT03086226) [7]. The trial aimed to establish an effective dosing regimen for fosravuconazole in combination with surgical lesion management, evaluating weekly doses of 200 and 300 mg over a 12-month period, marking the first use of fosravuconazole in such an extended treatment course.

Fosravuconazole is a water-soluble prodrug that is rapidly converted in vivo to the active compound ravuconazole. It was developed to enhance the solubility and oral bioavailability of ravuconazole. Fosravuconazole can be taken without food; however, coadministration with a high-fat meal has been shown to increase systemic exposure to ravuconazole [8, 9]. Ravuconazole offers broad-spectrum antifungal activity and has an extended half-life of over 1 week [10]. It demonstrates high protein binding (98%), extensive tissue distribution, and is predominantly metabolized in the liver by CYP enzymes, although data on its metabolism remain limited [11]. Ravuconazole has been well tolerated across various treatment regimens, with no significant toxicity reported in patients with eumycetoma receiving up to 12 months of therapy [7]. Additionally, it presents a low risk of drug-drug interactions involving CYP3A4, CYP2C9, and CYP2C19 [11]. In contrast, itraconazole's bioavailability varies with formulations and food intake. It is metabolized in the liver by CYP3A4 into its active metabolite, hydroxyitraconazole, with half-lives of 24 hours and 14 hours, respectively [12]. Both itraconazole and hydroxyitraconazole are highly lipophilic, over 99% protein-bound, widely distributed in tissues, and strong CYP3A4 inhibitors, potentially causing drug-drug interactions [13, 14].

The long half-life of ravuconazole allows once-weekly dosing and may improve cost-effectiveness and adherence compared to twice-daily itraconazole. Moreover, in vitro susceptibility studies show that ravuconazole has superior activity against *M. mycetomatis*, with a 90% minimum inhibitory concentration (MIC_90_) of 0.016 μg/mL, compared to 0.25 μg/mL for itraconazole [15]. However, the pharmacokinetics (PK) and pharmacodynamics (PD) of both ravuconazole and itraconazole in patients with eumycetoma remain unknown, which poses challenges to determining the optimal dose regimen.

This analysis aimed to characterize the PK of fosravuconazole, itraconazole, and hydroxyitraconazole in patients with eumycetoma, assessing the relationship between drug exposure and potential efficacy markers, such as MIC_90_ target attainment, lesion size reduction, and probability of complete cure, to inform the selection of a 200-mg or 300-mg dose of fosravuconazole for future clinical development.

## METHOD

### Study Design and Medication

The data originated from a single-center, randomized, double-blind, clinical superiority trial conducted at the Mycetoma Research Centre, Soba University Hospital, Sudan (NCT03086226) [7]. Patients with small-to-medium eumycetoma lesions requiring surgery were randomized 1:1:1 into 3 treatment arms: (1) fosravuconazole 300 mg (Eisai, Tsukaba, Japan) once daily on days 1–3, then on day 8, followed by weekly dosing for 12 months; (2) fosravuconazole 200 mg once daily on days 1–3, then on day 8, followed by weekly dosing for 12 months; or (3) the standard itraconazole (Sporanox, Janssen Pharmaceutica, Beerse, Belgium) 200 mg twice daily for 12 months. All drugs were administered orally on an unsupervised outpatient basis, with patients instructed to take the drugs with a full meal. Surgery was performed for all patients 6 months after the start of treatment. Investigation of the PK-PD of ravuconazole and itraconazole/hydroxyitraconazole was a secondary objective of the clinical trial.

### Pharmacokinetic Sampling, Bioanalysis, and Clinical Assessment

The schedule for PK sampling is summarized in Supplementary Table 1. Plasma concentrations of ravuconazole, itraconazole, and hydroxyitraconazole were quantified by Radboud University Medical Center using validated assays [7]. The validated ranges were 0.02–20 mg/L for ravuconazole, 0.05–9.82 mg/L for itraconazole, and 0.05–10.06 mg/L for hydroxyitraconazole.

Clinical assessment of eumycetoma lesion size was evaluated at baseline and at months 3, 6, 9, 12, and 15. The superficial lesion length and width were measured, and lesion surface area was calculated assuming an elliptical shape (Equation 1).


(1) $$Surface\;area\;(cm^{2})=\pi \;*\;0.25\;*\;Length\;(cm)\;*\;Width\;(cm)$$

Complete cure at 12 months end of treatment was defined based on the following criteria: absence of clinical evidence such as eumycetoma mass, sinus tract or discharge; normal ultrasonography or magnetic resonance imaging examination of the eumycetoma site; or if a mass was present, negative fungal culture from a surgical biopsy from the former eumycetoma site [7].

### Population Pharmacokinetic Analysis

Population PK analysis was conducted using NONMEM (version 7.5, ICON), Perl-speaks-NONMEM (PsN, version 5.0) [16], and Pirana (version 2.9.9) [17]. Data cleaning, data summary, graphical presentation, and statistical analysis were performed with R (version 4.2.3) software. Model parameters were estimated using the first-order conditional estimation with interaction method. Individual PK parameters were obtained by maximum a posteriori Bayesian estimation.

One-, two-, and three- compartment disposition models with first-order absorption and elimination were tested as structural PK models. Additionally, models incorporating Michaelis-Menten type saturable elimination from the central compartment were evaluated. Relative bioavailability (F) and fraction conversion of parent to metabolite (Fm) were assumed to be 1. All clearance (CL) and volume of distribution (Vd) parameters are therefore relative to F and Fm. To assess the effect of body size on the PK parameters, body weight was incorporated using an allometric function on all CL and Vd parameters. Fixed power exponents of 0.75 and 1 were applied to CL and Vd parameters, respectively, and were normalized to a standard body weight of 70 kg. Other potential covariate relationships, including age and sex, were first graphically explored and subsequently evaluated using a stepwise forward inclusion and backward elimination process.

Between-subject variability in PK parameters was assessed using an exponential variance model, assuming a log-normal distribution. Residual unexplained variability was tested using a proportional, additive, or combined proportional and additive error model. A joint parent-metabolite model was used to describe PK of itraconazole and hydroxyitraconazole. Between-occasion variability was tested on F, with each sampling day considered as a separate occasion, to account for random variability in fraction absorbed, drug adherence, and potential timing issues of the predose samples. Observations below the lower limit of quantitation (LLOQ) were excluded.

Secondary PK parameters were derived from the individual estimates obtained from the final PK models. Individual estimates of the area under the plasma concentration-time curve (AUC) from the start of treatment until 3, 6, and 12 months (AUC_0–3m_, AUC_0–6m_, and AUC_0–12m_) were calculated based on the individual empirical Bayes estimates of PK parameters. Dose proportionality between 200- and 300-mg fosravuconazole regimens was assessed by comparing the ratio of dose-corrected median predose concentrations measured at study days 8 and 15, and months 6, 9, and 12.

Model adequacy was evaluated based on physiological plausibility, graphical evaluation, and statistical significance. Statistical significance between hierarchical nested models was determined using the change in objective function value (OFV), calculated as minus 2 times the log-likelihood. Following a χ^2^ distribution, an OFV decrease of > 3.84 with 1 degree of freedom corresponded to a *P* value of < .05, which was considered statistically significant. Graphical evaluation was facilitated through standard goodness-of-fit plots. Additionally, visual predictive checks [18] and sampling importance resampling [19] were performed to assess predictive performance and parameter precision with 95% confidence intervals (CI) for the final model.

### Target Attainment and Pharmacokinetic-Pharmacodynamic Analysis

The individual percentage of time that the free drug concentration exceeded the in vitro MIC_90_ (% Time > MIC_90_) from start of treatment until end of treatment at 12 months was calculated for each drug using final individual empirical Bayes estimates of PK estimates. Drug concentrations were adjusted for protein binding to derive the free drug concentration. For ravuconazole, a protein binding value of 98% was used [11]. For itraconazole and hydroxyitraconazole, both the lowest and highest protein binding values (96% and 99%) reported in the literature were evaluated [14, 20]. The established in vitro MIC_90_ values were 0.016 mg/L for ravuconazole and 0.25 mg/L for itraconazole [15]. For hydroxyitraconazole, a similar MIC_90_ value was assumed as for the parent drug. Therefore, itraconazole and hydroxyitraconazole concentrations were added to assess attainment of MIC_90_ and conduct further PK-PD analysis.

Pharmacokinetic-pharmacodynamic relationships were examined from several perspectives. Firstly, the relationship between AUC_0–12m_ and probability of complete cure at the end of treatment was evaluated using logistic regression. Secondly, within each of the 3 treatment arms, the difference in drug exposure between cured and noncured patients was assessed using a Mann-Whitney *U* test. Thirdly, the effect of AUC_0–6m_ on the lesion size reduction (Equation 2) was evaluated using linear regression.


(2) $$\begin{array}{rl} & Change\;in\;lesion\;size\;(\text{\%})\\ & \;=\frac{Lesion\;size\;at\;6\;months\;(\;prior\;to\;surgery)-Base\;line\;lesion\;size}{Base\;line\;lesion\;size}\\ & \;\times 100 \end{array}$$

## RESULTS

### Patients and Sampling

**Figure 1.**
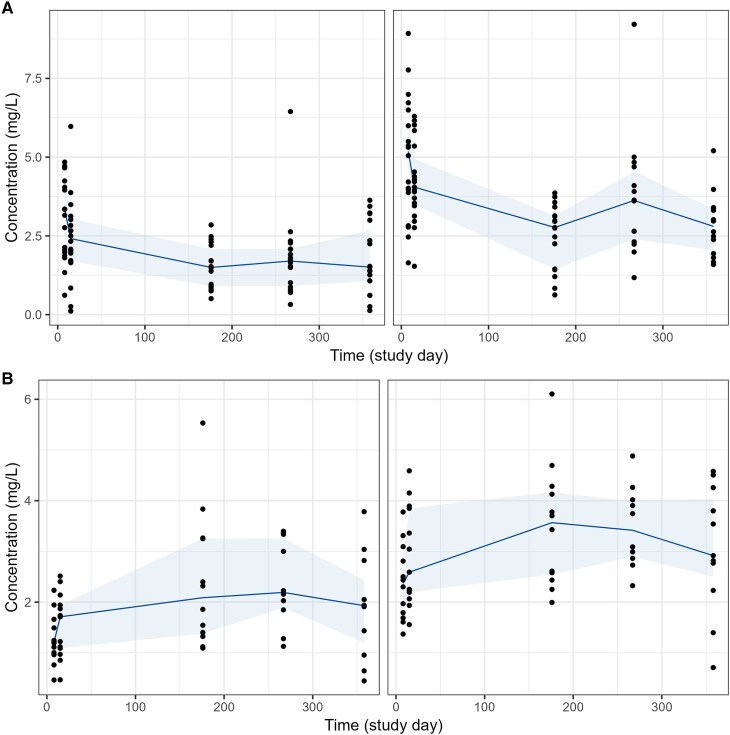
Predose plasma concentrations versus time. *A*, Measurement of ravuconazole in patients administered 200 mg (left) and 300 mg (right) fosravuconazole. *B*, Measurement of itraconazole (left) and hydroxyitraconazole (right) in patients administered itraconazole. The solid line represents the median value, while the shaded area indicates the interquartile range (25th to 75th percentiles).

A total of 52 patients from the per protocol population were included in this PK-PD analysis, 13 received 400 mg itraconazole, 20 received 200 mg fosravuconazole, and 19 received 300 mg fosravuconazole. Clinical characteristics of the study population are summarized in Table 1. Generally, demographics were similar between the 3 treatment arms, with similar weight, height, and age distributions. The predose concentration versus time profiles of ravuconazole and itraconazole/hydroxyitraconazole are depicted in Figure 1. The concentration versus time profiles from day 1 to day 22 and the intensive PK sampling at 3 and 6 months are shown in Supplementary Figures 1 and 2. A total of 544 ravuconazole, 175 itraconazole, and 180 hydroxyitraconazole concentrations were available for PK analysis, with 2 measurements below LLOQ for each analyte. The 24–96-hour postdose samples were unavailable for a large portion of patients, and none of the samples scheduled at 15 months were available.

**Table 1.** Demographics of Patients Available for the Pharmacokinetic Analysis

Characteristics	Treatment Arm		
	Fosravuconazole 200 mg (n = 20)	Fosravuconazole 300 mg (n = 19)	Itraconazole 400 mg (n = 13)
Age, y, median (IQR)	24.0 (20.0–29.0)	30.0 (25.5–37.5)	22.0 (19.0–27.0)
Height, m, median (IQR)	1.72 (1.62–1.77)	1.70 (1.67–1.74)	1.71 (1.60–1.77)
Weight, kg, median (IQR)	56.3 (51.8–65.6)	60.0 (55.0–67.0)	67.0 (51.0–72.0)
Male, n (%)	16 (80.0)	18 (94.7)	9 (69.2)
Lesion size, cm^2^, median (IQR)			
Baseline	11.7 (7.77–20.8)	17.0 (12.7–25.5)	12.5 (4.90–25.1)
Before surgery	6.17 (3.06–16.7)	16.7 (6.13–29.4)^a^	10.8 (4.08–15.8)
Treatment outcome, n (%)			
Cured	17 (85.0)	12 (63.2)	9 (69.2)
Not cured	3 (15.0)	7 (36.8)	4 (30.8)

Fosravuconazole 200 mg arm and Fosravuconazole 300 mg arm received once-daily dose on days 1–3, then on day 8, followed by weekly dosing for 12 mo. Itraconazole 400 mg arm received 200 mg twice-daily dose for 12 mo.

Abbreviation: IQR, interquartile range.

^a^Data available in 17 individuals.

### Pharmacokinetics of Ravuconazole

The median predose concentration of ravuconazole on study day 8 was higher than that on study day 15 (Supplementary Table 2), suggesting increased drug exposure as a result of the loading dose strategy, which slowly returned to steady-state. Throughout the treatment period, the median dose-corrected predose concentration ratios between the 300- and 200-mg regimens (300 mg/200 mg) were 1.03, 1.12, 1.23, 1.42, and 1.24, on study days 8 and 15, and months 6, 9, and 12, respectively, suggesting saturable clearance of ravuconazole in the observed dose range. Therefore, a model with Michaelis-Menten elimination was required to improve the underpredictions at higher concentrations.

**Figure 2.**
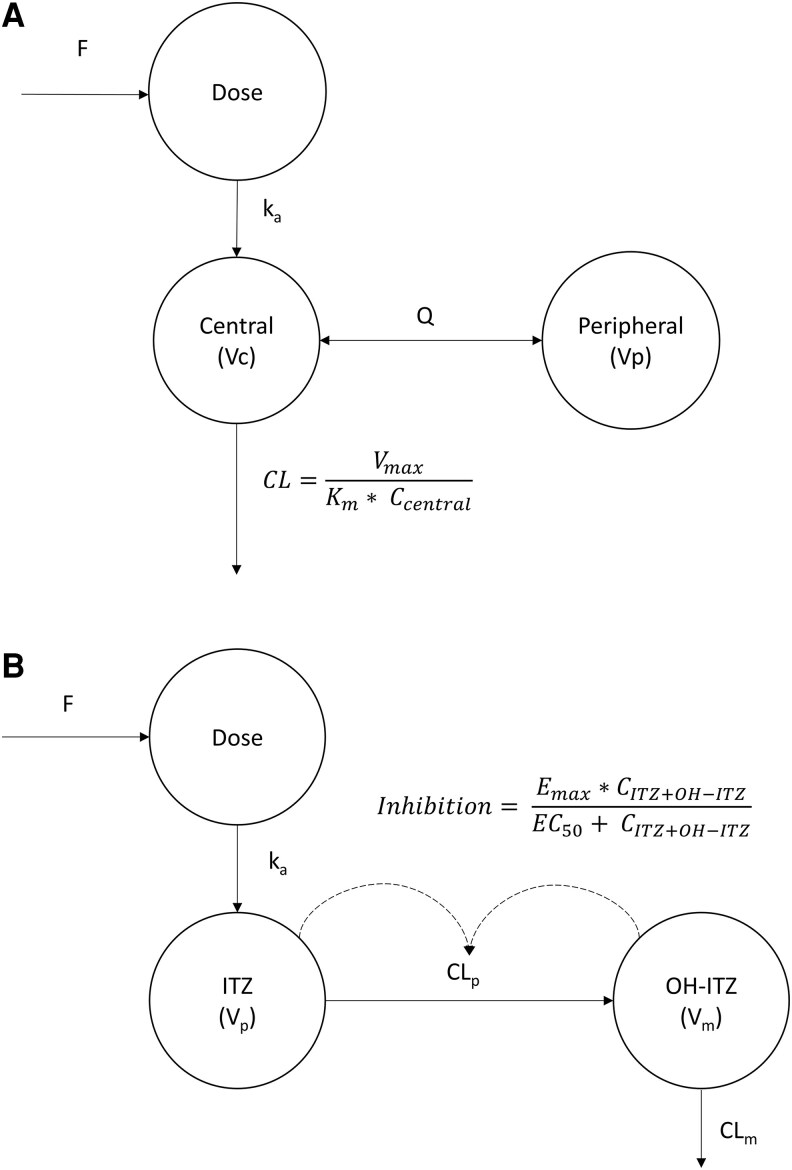
Final pharmacokinetics model. *A*, Ravuconazole. *B*, Itraconazole and hydroxyitraconazole. Abbreviations: C_central_, concentration of the central compartment; CL, clearance; CL_m_, clearance of metabolite OH-ITZ; CL_p_, clearance ITZ; EC_50_, itraconazole plus hydroxyitraconazole concentration (C_ITZ + OH-ITZ_) at 50% of autoinhibition effect; E_max_, maximum effect of autoinhibition; F, relative bioavailability; ITZ, itraconazole; k_a_, rate of absorption; K_m,_ Michaelis-Menten constant; OH-ITZ, hydroxyitraconazole; Q, intercompartmental clearance; Vc, volume of distribution central compartment; V_m_, volume of distribution for metabolite OH-ITZ; V_max_, maximum velocity; V_p_, volume of distribution.

The PK of ravuconazole was best described by a 2-compartment model with first-order absorption and Michaelis-Menten elimination from the central compartment (Figure 2), in which the maximum velocity (V_max_) was estimated at 4.55 mg/h (95% CI, 4.21–4.9). For stability reasons, the Michaelis-Menten constant (K_m_) was evaluated through sensitivity analysis, in the range of 2.5–6 mg/L based on observed peak concentration, and fixed at 4 mg/L in the final model. During the model building process, ravuconazole exposure during the loading dose phase was consistently underestimated, particularly among patients receiving the 300-mg regimen. To address this, an empirical increase in F during the loading dose phase was included, with an estimate of 1.63-fold (95% CI, 1.38–1.9), resulting in a 65-unit OFV decrease (*P* < .001).

In a typical patient (60 kg) receiving a dose of 200 mg or 300 mg, the median CL at steady state was either 0.71 L/h or 0.57 L/h, respectively. The rate of oral absorption (k_a_) was 0.0796 1/h (95% CI, .0526–.118), reflecting an absorption half-life of 8.7 hours. The Vd for the central and the peripheral compartments were 20.8 L (95% CI, 12.2–33.3) and 262 L (95% CI, 207–332), respectively. The parameter estimates of the final PK model are summarized in Table 2 and model evaluations are provided in Supplementary Figures 3 and 4.

**Table 2.** Population Pharmacokinetics Parameters

Parameter	Unit	Estimate	95% CI^a^
Fosravuconazole			
Rate of absorption (k_a_)	1/h	0.0796	.0526–.118
Maximum velocity (V_max_)^b^	mg/h	4.55	4.21–4.9
Michaelis-Menten constant (K_m_)	mg/L	4 (fixed)	…
Volume of distribution central compartment (Vc)^b^	L	20.8	12.2–33.3
Intercompartmental clearance (Q)^b^	L/h	9.03	6.32–12.8
Volume of distribution peripheral compartment (Vp)^b^	L	262	207–332
Fold increase in F during the loading dose phase^c^	…	1.63	1.38–1.9
Between-subject variability in Vc	CV%	190	119–381
Between-subject variability in K_m_	CV%	36	26.2–47
Between-occasion variability in F	CV%	11.6	6.29–15.6
Residual proportional error	CV%	41.7	38.2–45.7
Itraconazole and hydroxyitraconazole			
Rate of absorption (k_a_)	1/h	0.00376	.00328–.00429
Clearance of itraconazole (CL_p_)^b^	L/h	10.7	9.25–12.7
Volume of distribution for itraconazole (V_p_)^b^	L	25.5	13.6–43.1
Clearance of metabolite hydroxyitraconazole (CL_m_)^b^	L/h	5.16	4.56–5.91
Volume of distribution for metabolite hydroxyitraconazole (V_m_)^b^	L	4.85	2.09–8.06
Maximum effect of autoinhibition (E_max_)	…	1 (fixed)	…
Itraconazole plus hydroxyitraconazole concentration at 50% of autoinhibition effect (EC_50_)	mg/L	19.3	13.6–24.2
Between-subject variability in CL_p_	CV%	13.9	8.9–19.3
Between-subject variability in V_p_	CV%	122	74.5–221
Between-subject variability in CL_m_	CV%	19.1	10.9–26.1
Between-occasion variability in F	CV%	34.2	28.6–41.5
Residual proportional error, itraconazole	CV%	21.6	19–24.8
Residual proportional error, hydroxyitraconazole	CV%	13.8	12.1–16.1

All clearance and volume parameters are relative to relative bioavailability (F) and fraction metabolism (Fm), both assumed to be 1. CV%, coefficient of variation, was calculated by , $\sqrt{e^{\omega^{2}}-1}$ where ω^2^ is the variance of the random effect.

^a^Parameter precision (95% confidence interval [CI]) obtained using the sampling importance resampling (SIR) algorithm as implemented in Perl-speaks-NONMEM [16, 19].

^b^Allometric scaling based on body weight with a power exponent of 0.75 for all clearance and 1 for all volumes of distribution. Estimates are provided for a standardized body weight of 70 kg.

^c^Fosravuconazole loading doses were given on study days 1, 2, and 3.

### Pharmacokinetics of Itraconazole and Hydroxyitraconazole

The predose concentrations of hydroxyitraconazole were 1.5 to 1.8-fold higher compared to the parent drug, indicative of rapid metabolism and a potential first-pass effect (Supplementary Table 2). The structure of the final PK model of itraconazole and hydroxyitraconazole is depicted in Figure 2. Initially, a linear metabolism model overestimated itraconazole while underestimated hydroxyitraconazole concentrations in the first week, indicating saturable metabolism. Autoinhibition of itraconazole metabolism, likely due to CYP3A4 inhibition, was identified, leading to a 5.6-unit reduction in OFV and a substantial improvement in model misspecifications. This effect was modeled using a sigmoidal maximum effect (E_max_) function, with E_max_ fixed at 1 and the concentration required for 50% the maximum inhibition (EC_50_) was estimated at a combined itraconazole and hydroxyitraconazole concentration of 19.3 mg/L (95% CI, 13.6–24.2).

In the final population PK model for itraconazole and hydroxyitraconazole, the CL values were estimated to be 10.7 L/h (95% CI, 9.25–12.7) and 5.16 L/h (95% CI, 4.56–5.91), respectively, while the Vd values were 25.5 L (95% CI, 13.6–43.1) and 4.85 L (95% CI, 2.09–8.06), respectively. The parameter estimates of the final population PK model are summarized in Table 2 and model evaluations are provided in Supplementary Figures 5 and 6.

### Target Attainment and Pharmacokinetic-Pharmacodynamic Analysis

**Figure 3.**
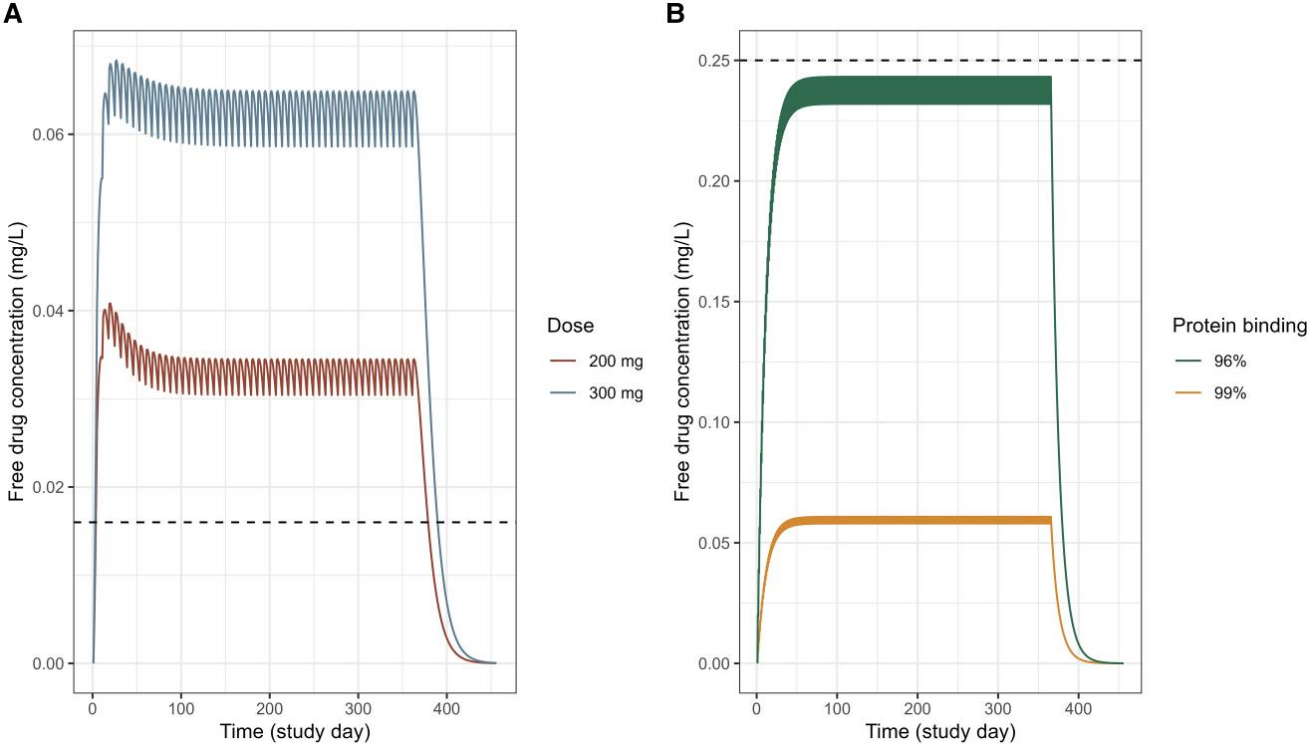
Estimated free drug concentrations of ravuconazole and itraconazole plus hydroxyitraconazole versus in vitro MIC_90_. *A*, The dashed lines represent MIC_90_ of 0.016 mg/L for ravuconazole [15]. The solid lines represents free drug concentration of ravuconazole (98% protein binding) with dose of fosravuconazole 200 mg (red) and 300 mg (blue). *B*, The dashed lines represent MIC_90_ of 0.25 mg/L for itraconazole and hydroxyitraconazole [15]. The solid lines represents free drug concentration of combined itraconazole and hydroxyitraconazole with 96% (green) and 99% (orange) protein binding. Abbreviation: MIC_90_, 90% minimum inhibitory concentration.

Free ravuconazole concentrations remained above the in vitro MIC_90_ value throughout the entire 12-month treatment for both the 200- and 300-mg ravuconazole regimens, with 92% (36/39) of patients achieving 100% Time > MIC_90_. No difference in % Time > MIC_90_ were observed between cured and noncured patients. Free itraconazole plus hydroxyitraconazole concentrations were below the in vitro MIC_90_ value, resulting in a 0% Time > MIC_90_ in all patients for 99% protein binding, and a median of 24% (interquartile range, 10%–81%) for 96% protein binding. To illustrate this, free drug concentrations in relation to MIC_90_ values in a typical patient are depicted in Figure 3.

**Figure 4.**
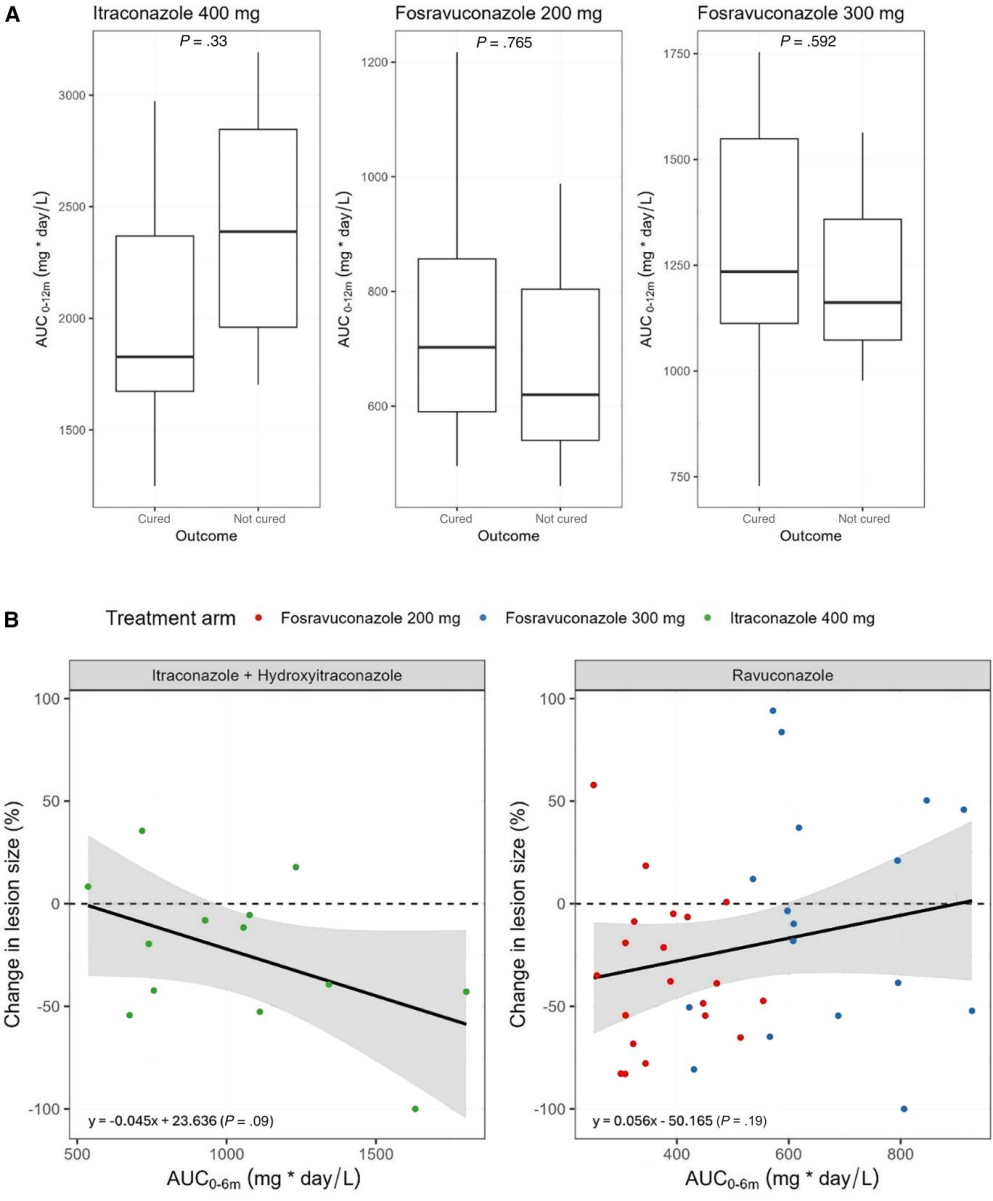
Exposure-response relationships. *A*, Drug exposure versus complete cure at the end of treatment at 12 months based on individual treatment arms. The horizontal line within the box represents the median. The box indicates the IQR, spanning from the 25th to the 75th percentile. Whiskers extend to the most extreme data points within 1.5 times the IQR from the lower and upper quartiles. Complete cure was defined as absence of clinical evidence such as eumycetoma mass, sinus tract, or discharge; normal ultrasonography or magnetic resonance imaging examination of the eumycetoma site; or if a mass was present, negative fungal culture from a surgical biopsy from the former eumycetoma site [7]. *B*, Drug exposure versus the change in lesion size prior to surgery from baseline. The x-axis shows the AUC_0–6m_ for ravuconazole and itraconazole plus hydroxyitraconazole up to the 6-month surgical intervention. The y-axis represents the percentage change in lesion size relative to baseline, with negative values indicating a decrease and positive values indicating growth. The solid line represents the fitted linear regression model. The shaded area indicates the 95% confidence interval for the estimated regression line. Abbreviation: AUC, area under the curve; IQR, interquartile range.

The secondary PK parameters derived from the final PK models are summarized in Table 3. For ravuconazole, the 300-mg regimen resulted in a higher than dose-proportional increase, leading to a 1.75-fold higher AUC_0–12m_ compared to the 200-mg regimen. Despite the large range of individual drug exposures, there was no correlation between AUC_0–12m_ and clinical cure at the end of treatment, for either patients on fosravuconazole or itraconazole (Supplementary Table 3). One patient who received fosravuconazole 200 mg and another who received 300 mg experienced lesion regrowth (< 2 cm^2^) at 15 months and 12 months, respectively. Neither patient exhibited lower AUC_0–12m_ values. When stratified by treatment arm, a slight trend towards higher AUC_0–12m_ values was observed for cured patients compared to noncured patients in both the 200- and 300-mg fosravuconazole cohorts (Figure 4*A*). However, these differences were not statistically significant. There was no significant effect of AUC_0–6m_ on the reduction in lesion size prior to surgery at 6 months for either fosravuconazole or itraconazole (Figure 4*B* and Supplementary Table 4). Consequently, based on the available data, no definitive PK-PD relationship could be established for either drug.

**Table 3.** Drug Exposure and Target Attainment of Ravuconazole, Itraconazole, and Hydroxyitraconazole From Study Start Until 3, 6, and 12 Months

	Treatment Arm and Compound			
	Fosravuconazole 200 mg (n = 20)	Fosravuconazole 300 mg (n = 19)	Itraconazole 400 mg (n = 13)	
	Ravuconazole	Ravuconazole	Itraconazole	Hydroxyitraconazole
Drug exposure, mg*day/L				
AUC_0–3m_	189 (163–227)	341 (298–406)	144 (116–190)	257 (205–292)
AUC_0–6m_	360 (308–448)	608 (568–795)	399 (271–519)	659 (470–717)
AUC_0–12m_	675 (587–867)	1180 (1090–1500)	784 (629–1110)	1270 (1080–1490)
Target attainment				
%Time > MIC_90_	100 (100–100)	100 (100–100)	24^a^ (10–81)	

Exposure metrics were derived based on individual empirical Bayes PK estimates of the final PK models. All values are represented as median (interquartile range). AUC_0–3m_, AUC_0–6m,_ and AUC_0–12m_ represent plasma concentration-time curves over 3, 6, and 12 mo, respectively. % Time > MIC_90,_ percentage of time that the free drug concentration exceeded the in vitro MIC_90_ from start of treatment until 12 mo. MIC_90_ values were 0.016 mg/L for ravuconazole and 0.25 mg/L for itraconazole and hydroxyitraconazole [15]. Protein binding was 98% for ravuconazole and 96%–99% for itraconazole and hydroxyitraconazole.

Abbreviations: AUC, area under the curve; MIC_90_, 90% minimum inhibitory concentration; PK, pharmacokinetics.

^a^Itraconazole and hydroxyitraconazole concentrations were combined for MIC_90_ assessment, using 96% as protein binding.

## DISCUSSION

To our knowledge this is the first PK study in patients with eumycetoma. It explores the population PK of ravuconazole, itraconazole, and hydroxyitraconazole in patients with eumycetoma, revealing nonlinear PK characteristics. Ravuconazole concentrations following fosravuconazole administration showed a more than dose-proportional increase. However, no relationship between drug exposure and lesion size reduction or probability of complete cure was found for either ravuconazole or itraconazole.

The 300-mg fosravuconazole regimen led to 75% higher AUC_0–12m_ than 200-mg regimen for a 50% dose increase (Table 3), but this did not translate into greater lesion reduction or cure rates (Figure 4). These results suggest that the 200-mg regimen is sufficient to achieve maximal response. Given similar efficacy and safety profiles, the 200-mg dose may be preferred for future implementation due to lower pill burden and cost-effectiveness. Similarly, no significant relationships were observed between itraconazole/hydroxyitraconazole exposure and clinical outcomes. These findings suggest that factors other than direct drug effect, such as surgical efficiency, play an important role in treating eumycetoma.

This study found decreased clearance and increased bioavailability of ravuconazole at higher concentrations, aligning with previous PK studies in rabbits showing nonlinear PK at higher dosages [21]. The saturable Michaelis-Menten elimination and increased bioavailability at loading doses are likely due to CYP3A saturation in the gastrointestinal tract and liver [22, 23]. However, previous clinical studies in Chagas disease (200–400 mg/week for 4–8 weeks) [24], onychomycosis (200 mg/day, 100–400 mg/week for 12 weeks) [25], and patients undergoing allogeneic stem cell transplantation (400–800 mg/day for 2 days pre- and posttransplantation) [11], did not report nonlinear PK for ravuconazole, suggesting factors like patient population, disease state, dosing, and treatment duration may influence its PK characteristics.

The population PK of itraconazole and hydroxyitraconazole have been studied in healthy individuals and patients with fungal infections [26–30], but direct comparisons are challenging due to variability in formulations, study populations, and modeling approaches. This study identified autoinhibition of itraconazole clearance by itraconazole and hydroxyitraconazole, suggesting saturable hepatic conversion, consistent with their roles as strong CYP3A inhibitors [13]. Similar autoinhibition was observed in a PK study in patients with talaromycosis [30]. Due to the small study population and data sparseness, particularly in the absorption phase, further studies with larger, more diverse cohorts are needed for validation.

Model estimates showed that free ravuconazole plasma concentrations remained above the in vitro MIC_90_ throughout the 12-month treatment, whereas free itraconazole and hydroxyitraconazole concentrations barely reached MIC_90_ (Table 3 and Figure 3) [15]. Despite similar cure rates and lesion reductions in patients treated with both drugs [7], these findings question the relevance of using in vitro MIC_90_ of itraconazole as a PD target, especially given the high plasma protein binding of itraconazole and its metabolite, which may affect translating in vitro activity to clinical outcomes. Additionally, lesion size assessment was limited to superficial measurements, overlooking lesion depth, which may introduce bias. In the future, standardized imaging techniques like ultrasound or magnetic resonance imaging should be used to provide a more accurate assessment of lesion mass, and noninvasive markers for antifungal treatment response should be developed as potential PD surrogate end points.

In conclusion, this study characterized the population PK of ravuconazole, itraconazole, and hydroxyitraconazole in patients with eumycetoma. Ravuconazole displayed decreased clearance and increased bioavailability at higher concentrations, suggesting metabolic enzyme saturation, while itraconazole showed reduced clearance due to autoinhibition. No significant correlations were found between exposure to ravuconazole or itraconazole and the reduction in lesion size or the probability of achieving complete cure. The 200 mg fosravuconazole dose may be preferred over 300 mg for its lower pill burden and cost-effectiveness. Further research should explore combination therapies, with or without surgery, to reduce treatment duration, as well as appropriate biomarkers and noninvasive imaging techniques to assess antifungal treatment response.

## Supplementary Material

jiaf279_Supplementary_Data

## Supplementary Data

Supplementary materials are available at *The Journal of Infectious Diseases* online (http://jid.oxfordjournals.org/). Supplementary materials consist of data provided by the author that are published to benefit the reader. The posted materials are not copyedited. The contents of all supplementary data are the sole responsibility of the authors. Questions or messages regarding errors should be addressed to the author.

## Notes

***Acknowledgments.*** We extend our deepest gratitude to the patients who participated in this study, as well as their families and communities, whose contributions were invaluable to the success of this research. We also sincerely thank the coinvestigators, clinical trial monitors, nurses, laboratory staff, and the entire team at the Mycetoma Research Center for their dedication and support throughout the study. Additionally, we are especially grateful to Louise Burrows (Drugs for Neglected Diseases Initiative) for her thoughtful and thorough review of the manuscript.

***Author contributions.*** W. C. and T. D. performed the analysis and drafted the manuscript. A. H. F., N. S. W., E. E. Z., and E. S. W. M. designed the clinical study. E. S. A., S. M. B., O. E. B., L. A. F., A. A. M., M. E. N. B., H. Y. A., E. E. S., N. A. M., A. M. M., P. O., and T. E. collected the data. R. J. B. measured the PK samples. K. H. oversaw the use of fosravuconazole. B. A. N. and F. A. supervised practical aspects and quality control. All authors reviewed and approved the final manuscript.

***Financial support.*** This work was supported by Drugs for Neglected Diseases initiative (DNDi), which received financial support from Global Health Innovative, Technology Fund, Japan (grant number G2017–106); Dutch Ministry of Foreign Affairs; Swiss Agency for Development and Cooperation; Republic and Canton of Geneva, International Solidarity Service, Switzerland; Médecins Sans Frontières International; Médecins Sans Frontières Switzerland; UK International Development; and other private foundations and individuals. T. D. was supported by the Swedish Research Council (grant number VR 2022–01251).
