# Supplementary material for: Pharmacokinetics and Pharmacodynamics of Fosravuconazole, Itraconazole, and Hydroxyitraconazole in Sudanese Patients With Eumycetoma
Source: J Infect Dis. 2025 May 28;232(3):e518–28. doi: 10.1093/infdis/jiaf279 (PMC12455320; doi:10.1093/infdis/jiaf279)

Supplementary Material

### Table S1. Pharmacokinetics sampling and clinical assessment schedule according the study protocol

|  | Time of treatment ^b^ | | | | | | | | |
| --- | --- | --- | --- | --- | --- | --- | --- | --- | --- |
| Time after dose ^a^ | Day 1 | Day 8 | Day 15 | Day 22 | 3 Months | 6 Months ^c^ | 9 Months | 12 Months | 15 Months |
| Pre-dose | X | X | X |  |  | X | X | X | X |
| 2-4 h post-dose ^b^ | X |  |  | X | X | X |  |  |  |
| 6-12 h post-dose ^b^ | X |  |  |  | X | X |  |  |  |
| 24-96 h post-dose ^b^ | X |  |  |  | X | X |  |  |  |
| ^a^ Time windows in which pharmacokinetic sampling should have been performed.  ^b^ Indicated time of treatment are nominal/scheduled time points for visits. Sampling windows were ± 3 days on Days 8, 15 and 22, and ± 7 days on 3 Months (Day 57), 6 Months (Day 176) and 12 Months (Day 358), and ± 14 days on 9 Months (Day 267) and 15 Months (Day 455).  ^c^ Plasma PK samples collection was performed prior to the surgery. | | | | | | | | | |

### Table S2. Pre-dose concentrations of ravuconazole, itraconazole and hydroxyitraconazole.

|  | Time of treatment ^a^ | | | | |
| --- | --- | --- | --- | --- | --- |
| Treatment arm | Day 8 | Day 15 | 6 Months | 9 Months | 12 Months |
| Fosravuconazole 200 mg |  |  |  |  |  |
| Ravuconazole (mg/L) | 3.35  [2.02,4.15] | 2.41  [1.71,3.04] | 1.50  [0.9,2.08] | 1.70  [0.91,2.08] | 1.51  [1.07,2.67] |
| Fosravuconazole 300 mg |  |  |  |  |  |
| Ravuconazole (mg/L) | 5.18  [3.90,6.37] | 4.05  [3.50,4.94] | 2.77  [1.45,3.14] | 3.62  [2.40,4.54] | 2.80  [2.05,3.36] |
| Itraconazole 400 mg |  |  |  |  |  |
| Itraconazole (mg/L) | 1.21  [0.99,1.53] | 1.71  [1.09,1.94] | 2.09  [1.38,3.25] | 2.19  [1.89,3.25] | 1.93  [1.19,2.43] |
| Hydroxyitraconazole (mg/L) | 2.37  [1.76,2.88] | 2.59  [2.19,3.85] | 3.57  [2.54,4.17] | 3.42  [2.89,3.99] | 2.92  [2.50,4.03] |
| All values are represented as median [IQR].  ^a^ Indicated time of treatment are nominal/scheduled time points for visits. Sampling windows were ± 3 days on Days 8 and 15, and ± 7 days on 6 Months (Day 176) and 12 Months (Day 358), and ± 14 days on 9 months (Day 267). | | | | | |

### Table S3. Results of logistic regression on cure at the end of treatment at 12 months.

|  | Estimate | Std. Error | p-value | Odds ratio (95%CI) |
| --- | --- | --- | --- | --- |
| **Ravuconazole (200 and 300 mg)** | | | | |
| AUC_0-12m_ (mg*day/L) | -0.0007 | 0.001 | 0.47 | 0.999 (0.997-1.00) |
| **Itraconazole + Hydroxyitraconazole** | | | | |
| AUC_0-12m_ (mg*day/L) | -0.0011 | 0.001 | 0.29 | 0.999 (0.997-1.00) |
| The AUC_0-12m_ of ravuconazole is evaluated without differentiating between the 200 mg and 300 mg treatment arms. The AUC_0-12m_ of itraconazole and hydroxyitraconazole were combined, assuming comparable impact on the studied outcome. | | | | |

### Table S4. Results of linear regression on percentage change in lesion size prior to surgery at 6 months.

|  | Estimate | 95% CI | Std. Error | p-value |
| --- | --- | --- | --- | --- |
| **Ravuconazole (200 and 300 mg)** | | | | |
| AUC_0-6m_ (mg*day/L) | 0.055 | -0.026-0.136 | 0.04 | 0.19 |
| **Itraconazole + Hydroxyitraconazole** | | | | |
| AUC_0-6m_ (mg*day/L) | -0.045 | -0.095-0.003 | 0.025 | 0.09 |
| The AUC_0-6m_ of ravuconazole is evaluated without differentiating between the 200 mg and 300 mg treatment arms. The AUC_0-6m_ of itraconazole and hydroxyitraconazole were combined, assuming comparable impact on the studied effect. | | | | |

### Figure S1. Ravuconazole concentration versus time

1. Study days 1 to 22


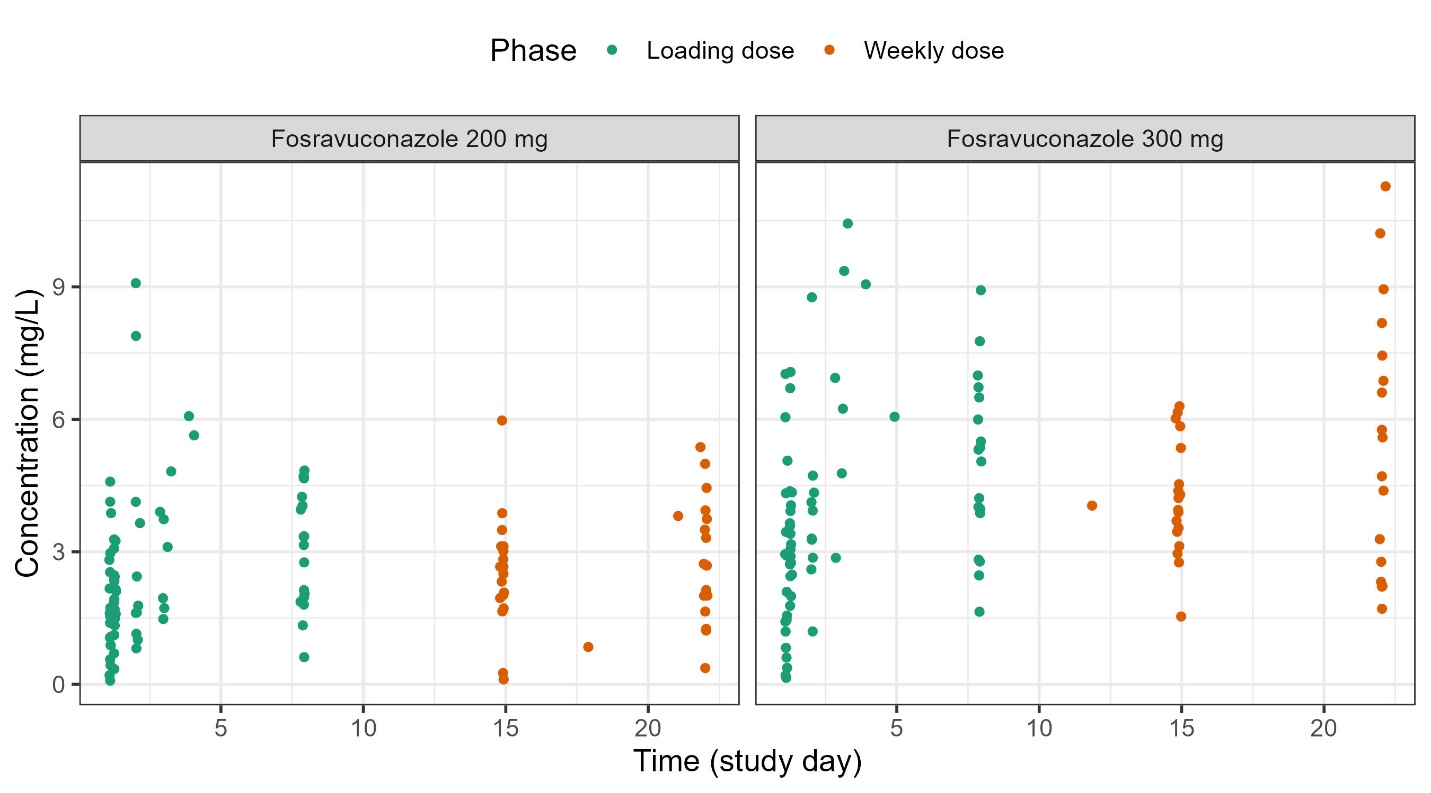


1. Intense PK sampling at months 3 (Day 85) and 6 (Day 176)


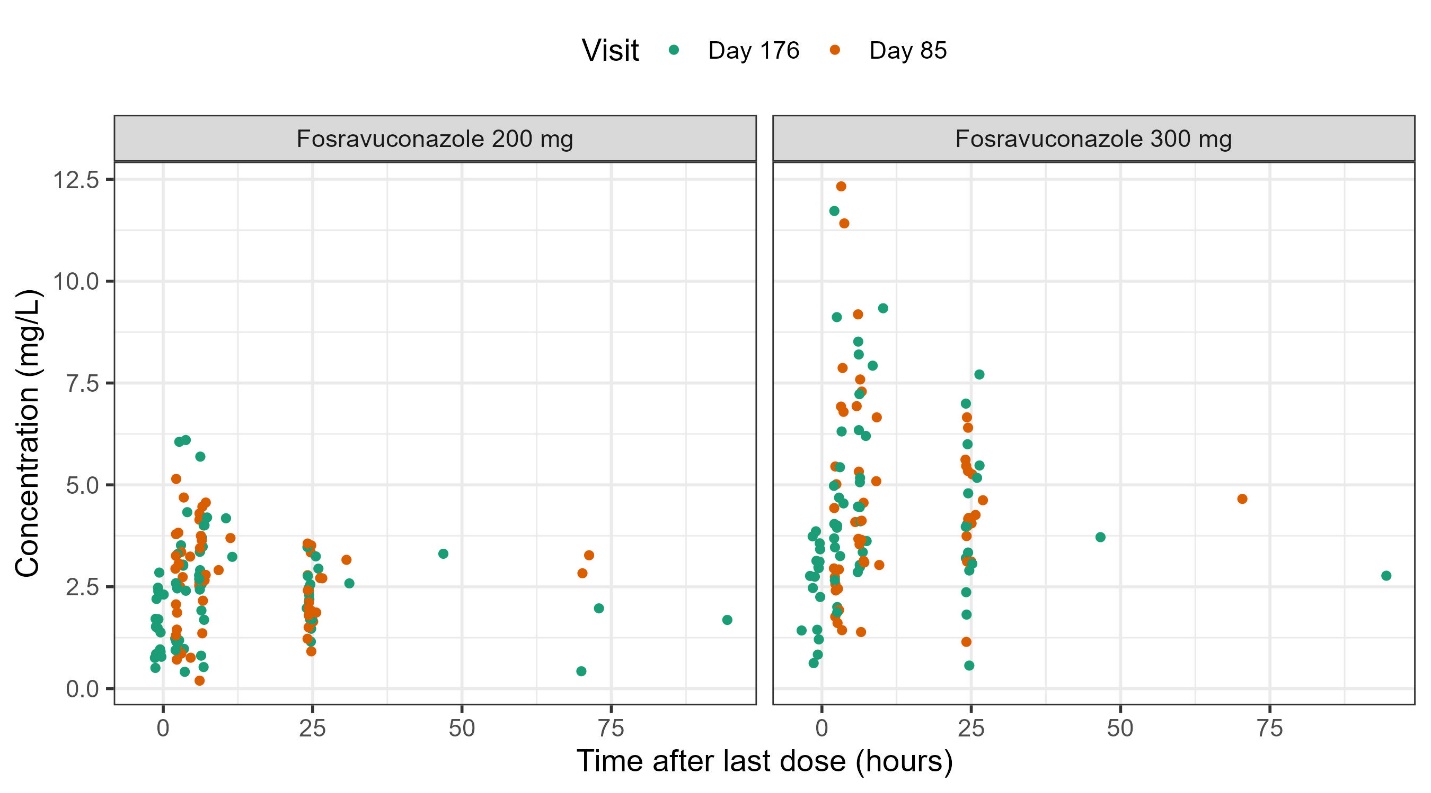


### Figure S2. Itraconazole and hydroxyitraconazole concentration versus time

1. Study day 1 to 22


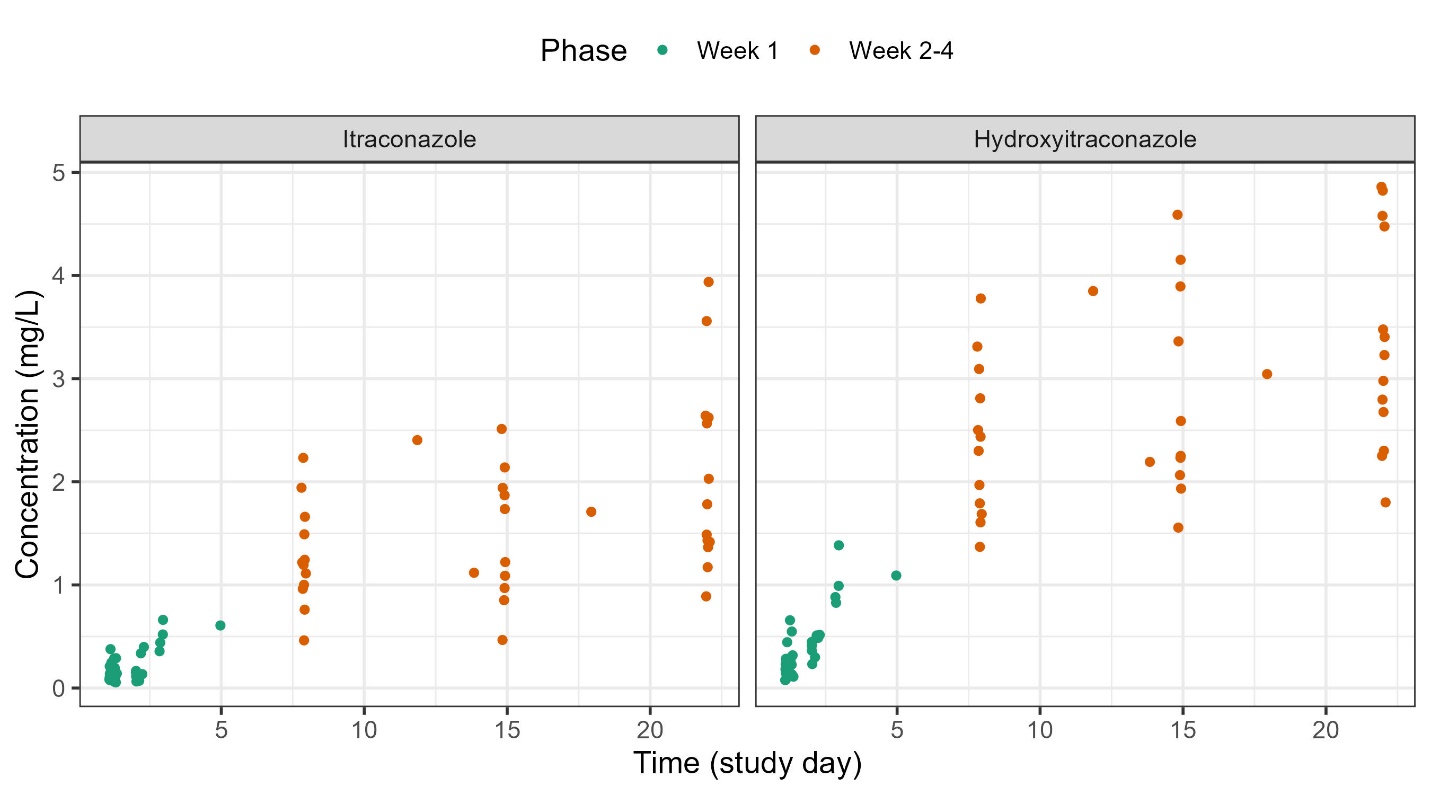


1. Intense PK sampling at months 3 (Day 85) and 6 (Day 176)


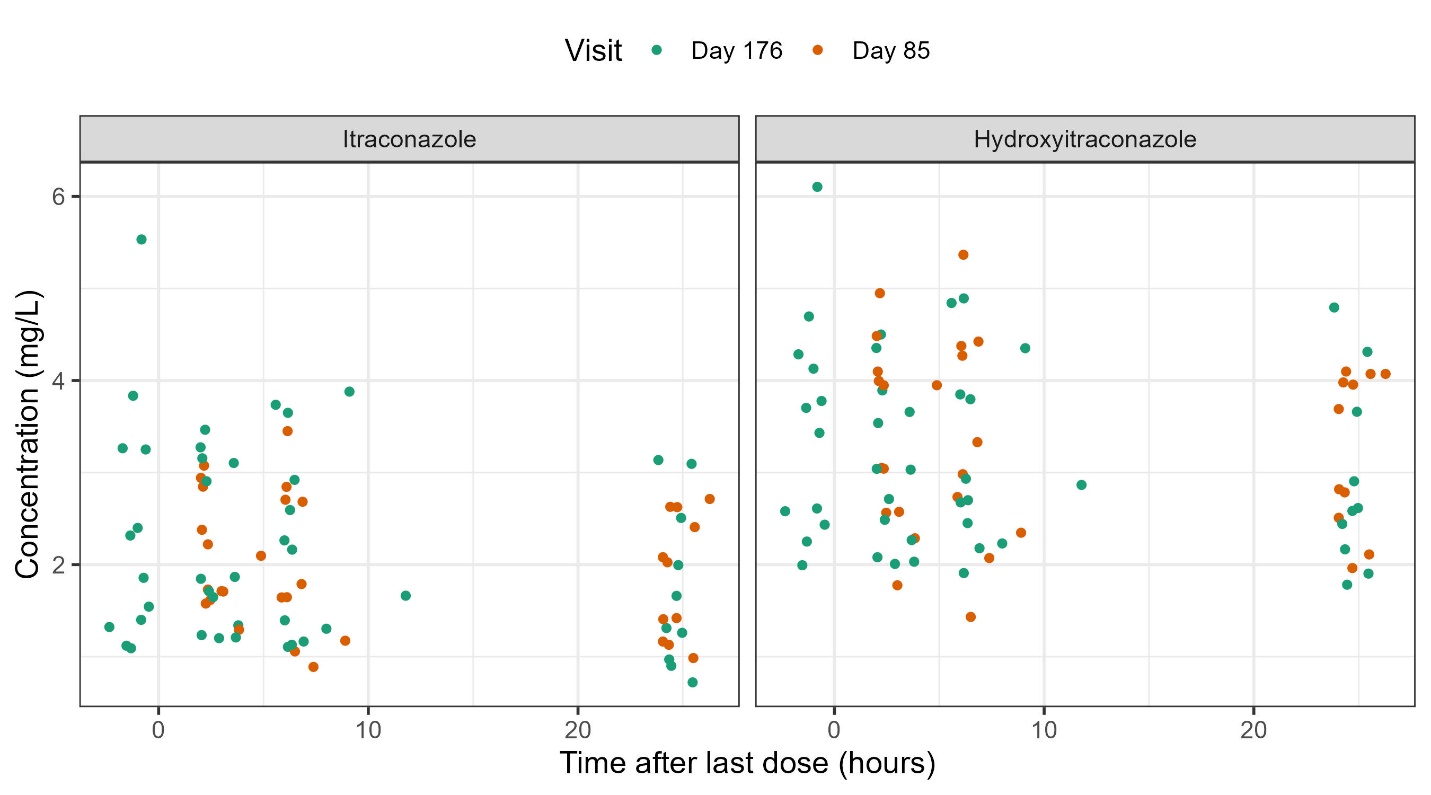


### Figure S3. Goodness-of-fit plots of ravuconazole final PK model.

DV, observations (mg/L); PRED, population model predictions (mg/L); IPRED, individual model predictions (mg/L); CWRES, conditional weighted residuals; TIME (day).


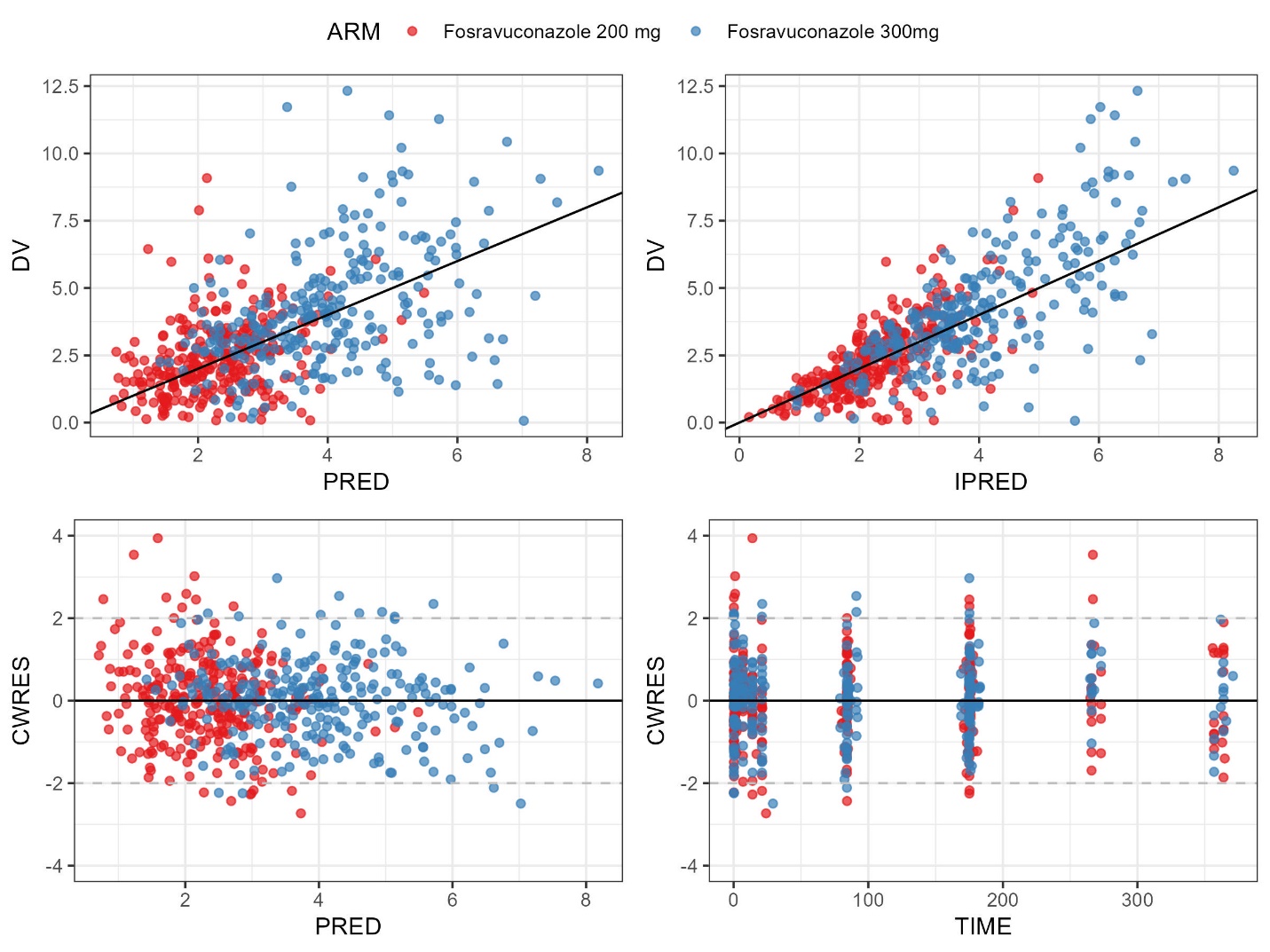


### Figure S4. Prediction corrected VPC of ravuconazole based on final model.

Black solid and dashed lines represent median and 50%-interval of the prediction corrected observations, red shaded area represents the 95%-confidence interval (CI) of the median prediction, and blue shaded area represents 95%-CI of the 25th and 75th prediction interval.


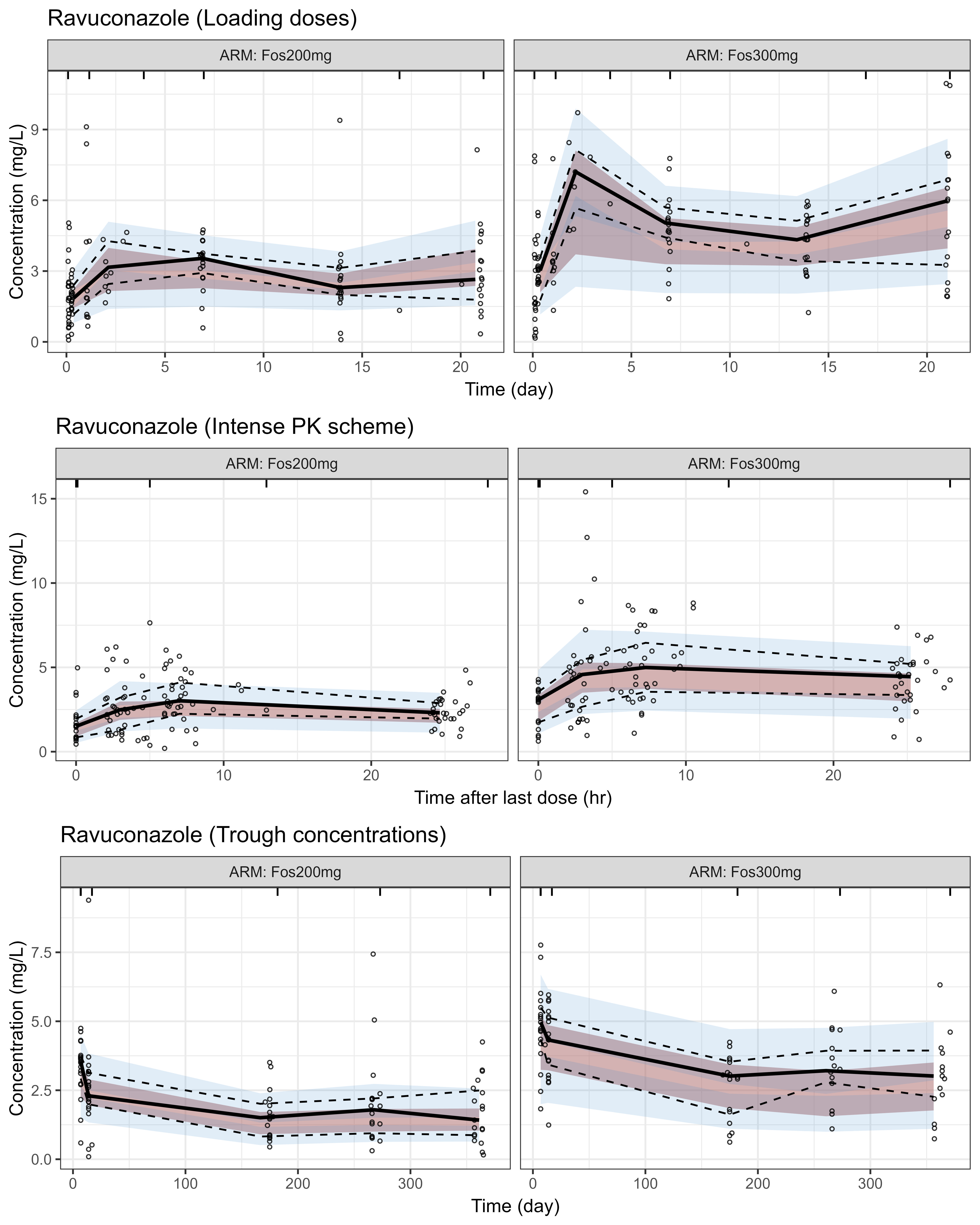


### Figure S5. Goodness-of-fit plots of itraconazole and hydroxyitraconazole final PK models.

DV, observations (mg/L); PRED, population model predictions (mg/L); IPRED, individual model predictions (mg/L); CWRES, conditional weighted residuals; TIME (day).


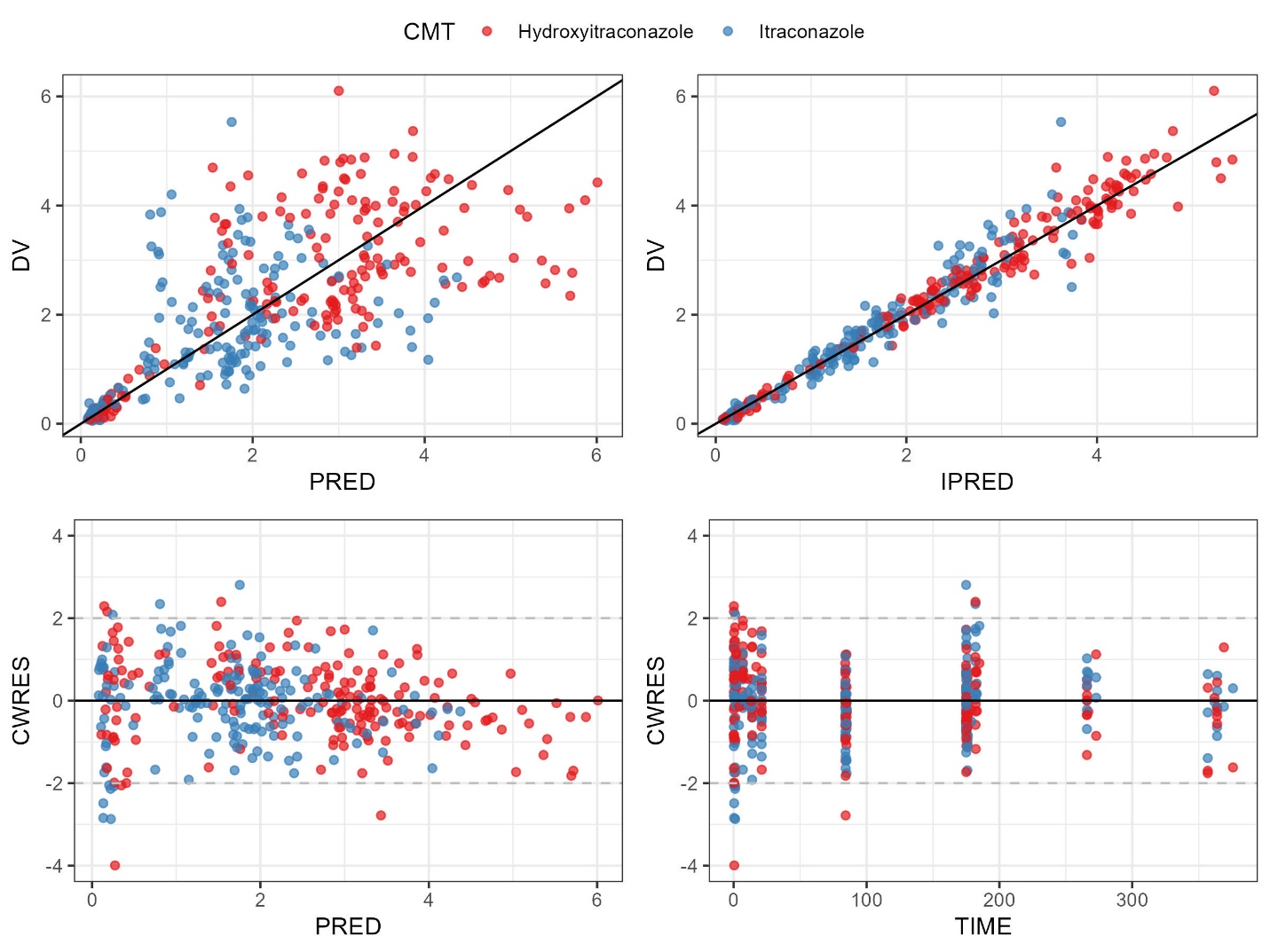


### Figure S6. Prediction corrected VPC of itraconazole and hydroxyitraconazole based on final model.

Black solid and dashed lines represent median and 50%-interval of the prediction corrected observations, red shaded area represents the 95%-confidence interval (CI) of the median prediction, and blue shaded area represents 95%-CI of the 25th and 75th prediction interval.


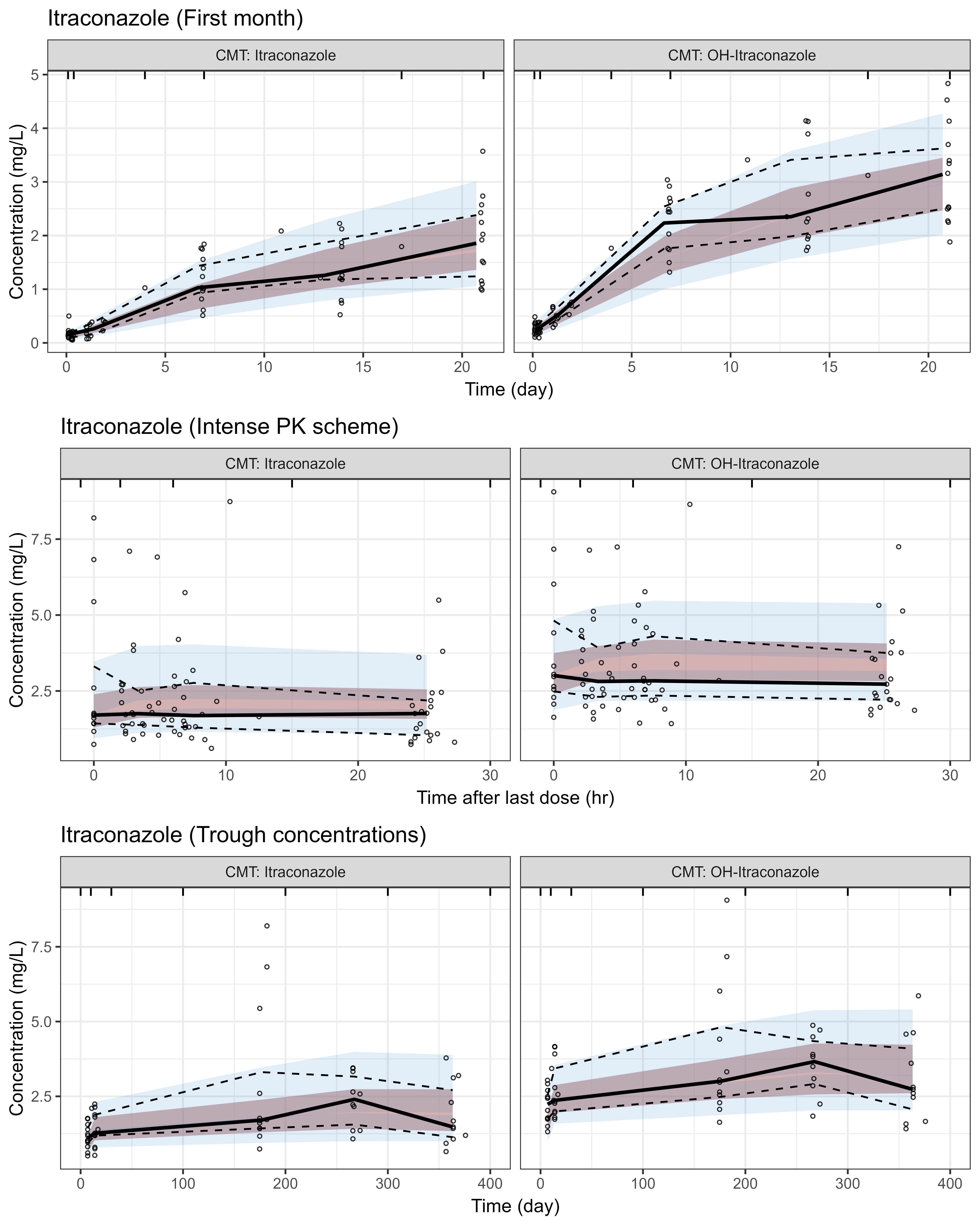

Supplement: jiaf279_Supplementary_Data [file jiaf279_supplementary_data.docx]
